# Optimal Cerebral Perfusion Pressure in Brain Injury: Physiological Relationships and Outcome

**DOI:** 10.1227/neu.0000000000003411

**Published:** 2025-04-03

**Authors:** Adam I. Pelah, Agnieszka Kazimierska, Marek Czosnyka, Gregory W. J. Hawryluk

**Affiliations:** * Department of Clinical Neurosciences, University of Cambridge, Cambridge, UK;; ‡ Department of Biomedical Engineering, Wroclaw University of Science and Technology, Wroclaw, Poland;; § Cleveland Clinic, Neurological Institute, Akron General Hospital, Fairlawn, Ohio, USA

**Keywords:** Brain injury, CPP_opt_, Intracranial hypertension, Neurosurgery, Perfusion, Trauma, Outcome, Oxygenation, Neuromonitoring

## Abstract

**BACKGROUND AND OBJECTIVES:**

The priority for measuring and optimizing physiological metrics in brain injury care remains to be determined. Calculating and targeting optimal cerebral perfusion pressure (CPP_opt_) is an emerging treatment paradigm, but its association with other parameters is uncertain. A previous analysis of 22 patients found that brain tissue oxygenation (P_bt_O_2_) peaked when CPP values were near CPP_opt_. This study sought to validate those findings using a distinct, larger cohort. It also studied the relationship between CPP_opt_ and physiological parameters related to intracranial dynamics and with neurological outcome.

**METHODS:**

P_bt_O_2_, intracranial pressure (ICP), and arterial blood pressure data were collected during a 15-year period from 432 brain injury patients at 4 cooperating trauma centers. CPP_opt_ was retrospectively computed.

**RESULTS:**

The median age was 36 years (*n =* 316), the median admission Glasgow coma score was 6 (*n* = 323), and 75% of the patients were men (*n* = 324). In aggregate data, P_bt_O_2_ peaked at CPP values near CPP_opt_ (+/− 2 mm Hg). Proportion of out-of-range ICP measurements (>22 mm Hg) and positive pressure reactivity index were higher in dying and unfavorable outcome groups, and increased with worsening outcome. Time spent near CPP_opt_ was significantly lower in dying patients but not in patients with unfavorable outcome. Time near CPP_opt_ was, however, correlated with better outcome. Proportion of out-of-range P_bt_O_2_ (<20 mm Hg) was not associated with outcome or mortality.

**CONCLUSION:**

The results verify CPP_opt_ as physiologically significant and that in aggregate data achievement of CPP_opt_ is associated with maximized P_bt_O_2_. Compliance with the ICP treatment threshold was, though, the only modifiable physiological variable associated with both functional outcome and mortality. Our results support optimization of ICP with highest priority. Further study is required in patients in whom CPP_opt_ is specifically targeted.

## Graphical abstract

**Figure:**
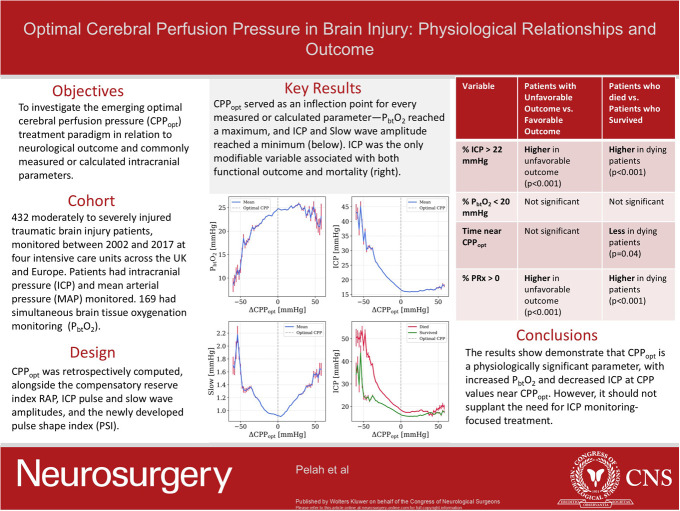

**ABBREVIATIONS:**
AMP: ICP pulse amplitude
ANOVA: analysis of variance
CPP_opt_: optimal cerebral perfusion pressure
MAP: mean arterial blood pressure
P_bt_O_2_: brain tissue oxygenation
PRx: pressure reactivity index
PSI: pulse shape index
RAP: R-amplitude pressure
TBI: traumatic brain injury.

Optimal cerebral perfusion pressure (CPP_opt_) was first introduced in 2002 as a method of assessing and optimizing cerebral autoregulation.^1^ CPP_opt_ is calculated over a fixed or variable interval, by determining the CPP at which the cerebrovascular pressure reactivity (expressed with the pressure reactivity index [PRx]) is most optimal.^1-3^ PRx is calculated as the moving correlation coefficient between slow waves of intracranial pressure (ICP) and mean arterial blood pressure (MAP); a positive PRx indicates dysfunctional autoregulation.^4,5^ CPP_opt_ can be targeted pharmacologically, the safety of which was investigated in the CPP_opt_ Guided Therapy: Assessment of Target Effectiveness trial, which found that matching a calculated CPP_opt_ was safe for brain-injured patients.^6-8^ In addition, previous literature has suggested that CPP above CPP_opt_ is associated with increased severe disability and that CPP below CPP_opt_ is associated with increased mortality.^9^ To date, the physiological relationship between CPP_opt_ and hemodynamics has primarily been examined through the lens of autoregulation.^10-12^ There have been few studies exploring how CPP_opt_ relates to other hemodynamic parameters or other treatment targets, such as brain tissue oxygenation (P_bt_O_2_) or ICP.^13-16^ Some centers target CPP_opt_ as a higher priority than the more established treatment paradigm of targeting the ICP treatment threshold.^17^ It has been uncertain which treatment approach is superior, or whether achieving CPP_opt_ improves other metrics such as P_bt_O_2_,^18^ or other indices characterizing cerebrospinal dynamics. Most important to understand is whether a monitoring modality is associated with improved clinical outcome. The metric most strongly associated with improved outcome should be targeted with highest priority. Recently published data from 22 traumatic brain injury (TBI) patients found a distinct bell-shaped relationship between ΔCPP_opt_ (defined as the difference between CPP and CPP_opt_) and P_bt_O_2._ This suggested, in aggregate data, that achieving CPP_opt_ was associated with optimization of P_bt_O_2_. As the achievement of CPP_opt_ was not associated with optimal P_bt_O_2_ in all patients and did not prevent all out-of-range P_bt_O_2_ (<20 mm Hg) values, however, the authors judged that targeting CPP_opt_ does not supplant the need for P_bt_O_2_ monitoring. The results supported the conclusion that CPP_opt_ is useful to target clinically but may not be sufficient for full optimization of brain physiology.^18^ It has been important to validate these findings in a larger independent patient cohort and to determine which metric is most strongly associated with clinical outcome to help establish monitoring and management priorities for neurocritical care. This study sets out to do this and includes an analysis of how CPP_opt_ is related to a range of ICP-derived physiological variables, which inform intracranial dynamics. Such variables include ICP pulse amplitude (AMP) and the compensatory reserve index R-amplitude pressure (RAP), used clinically as an indicator of reserve of pressure–volume compensation in TBI and hydrocephalus (**Supplemental Digital Content 1** [http://links.lww.com/NEU/E673]).^19-21^ The amplitude of slow waves of ICP (*Slow*) has been shown to be associated with vasogenic activity and patient outcome.^22-24^ Pulse shape index (PSI) uses machine learning models to categorize the ICP pulse waveform into a scale that reflects compliance-related changes in the waveform shape. PSI has been shown to be associated with outcome and the presence of midline shift and mass lesions in computed tomography examinations.^25,26^ In this study, we replicate, validate, and extend the aforementioned findings in an independent, larger patient cohort from several cooperating institutions. We sought primarily to determine how time spent near CPP_opt_ relates to patient outcomes (not reported in the previous study), and how CPP_opt_ relates to the achievement of other common physiological treatment targets such as ICP and P_bt_O_2._ These findings, as well as CPP_opt_'s relationship with the additional variables, have not been previously reported to our knowledge.

## METHODS

In total, 432 moderate to severely injured TBI patients were monitored between 2002 and 2012 2017, from 4 intensive care units: Addenbrooke's Hospital Cambridge, UK; Charité—Universitätsmedizin, Berlin; University Hospital North Norway, Norway; and São João University Hospital, Porto. Ethical clearance has been obtained at the time of monitoring separately in the 4 participating hospitals. Anonymized recordings were deposited in Cambridge Brain Physics Laboratory database for training purposes. Ethical approval for reanalysis and publishing of stored material was granted in Cambridge (REC 23/YH/0085). Consent was obtained from family or next of kin at time of monitoring. Patients were sedated, intubated, and mechanically ventilated. Treatment protocol was CPP/ICP oriented, with care taken to maintain CPP above 50 mm Hg and ICP below 20 mm Hg per the contemporaneous Brain Trauma Foundation guidelines.^27^ Data were collected at the bedside using the computerized ICM+ system (Cambridge Enterprise Ltd). P_bt_O_2_ was collected through the Licox (Integra LifeSciences) system, MAP through arterial line (Baxter Healthcare), and ICP through intraparenchymal probe (Codman MicroSensor or Raumedic Neurovent). Clinical outcome was collected at 6 months postinjury using the Glasgow Outcome Scale (GOS).^28^ Metrics related to recording time and measured values for studied patients are provided in **Supplemental Digital Content 2** (http://links.lww.com/NEU/E674).

Primary data analysis was performed using the ICM+ software. PRx was calculated as the moving correlation coefficient between averaged (10 seconds windows) ICP and MAP, with a 5-minute calculation window. CPP_opt_ was calculated as the minimum of the U-shaped plot of PRx vs CPP, with a 4-hour calculation window. CPP_opt_ was not computed or targeted during treatment. RAP was calculated as the moving correlation coefficient between AMP, obtained using the fundamental frequency of ICP, and mean ICP (10 seconds periods), with a 5-minute calculation window. *Slow* was calculated as the square root of the signal power with a 10-minute calculation window. PSI was calculated using a pretrained machine learning model for morphological classification of the ICP pulse waveform, with a 1-minute calculation window. ΔCPP_opt_ was obtained by subtracting CPP_opt_ from CPP. Calculated variables were updated every minute. “Near CPP_opt_” was defined as CPP values within 2 mm Hg of CPP_opt_. All variables were averaged over 1-minute intervals for further analysis in Python. To aid in eliminating artefactual data, ICP was restricted between 0 and 100 mm Hg, P_bt_O_2_ between 0 and 60 mm Hg, ΔCPP_opt_ between −60 and 60 mm Hg, *Slow* between 0 and 6, and AMP between 0 and 10 mm Hg.

### Statistical Analysis

Statistical analysis was performed in Python using the *SciPy* library. Analysis of variance (ANOVA) was first used to establish differences between means and proportions of variables in 3 groups: below, at, and above CPP_opt_. Post hoc tests between groups were performed with Student *t*-test, with a *P*-value of <.05 considered significant. Plots showing mean values against ΔCPP_opt_ were generated using binned mean values (bin width of 0.75 mm Hg). Analyses were also repeated for the proportion of measured physiological values outside of established ranges as defined by accepted treatment thresholds: >20 mm Hg for P_bt_O_2_ and <22 mm Hg for ICP.^27^

### Data availability

According to policy of each hospital, clinical recordings cannot be shared without additional approval of Ethical Committee. For details contact MC.

## RESULTS

The median age was 36 years (*n* = 316), and the median Glasgow coma scale was 6 (*n* = 323). 75% of the cohort were men (*n* = 324). One patient was excluded because of not having ICP recorded, and 15 were excluded because of their recordings being under 6 hours in length, rendering the CPP_opt_ calculation unreliable. This left 416 patients total in the analysis. CPP_opt_ could be calculated 51% of the total time, with patients autoregulating (PRx <0) 44% of the total time. Data were distributed normally across differing ΔCPP_opt_ values for all 6 calculated or measured parameters (**Supplemental Digital Content 3** [http://links.lww.com/NEU/E675]).

### Brain Tissue Oxygenation

**FIGURE 1.**
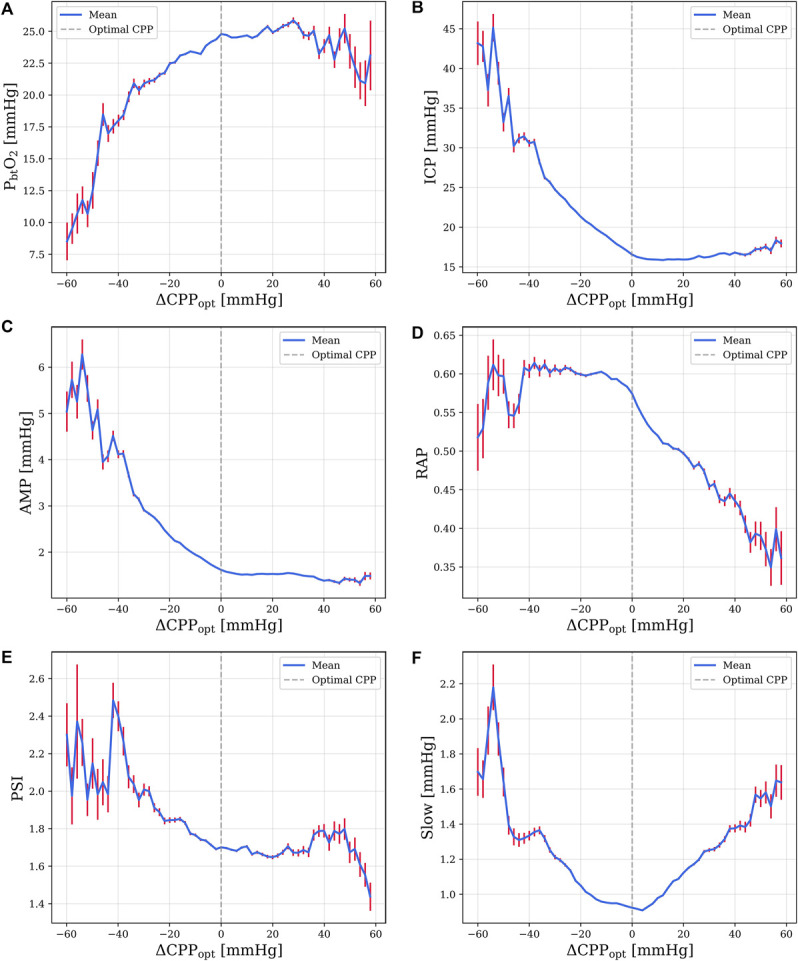
Mean values of intracranial parameters plotted against deviation from optimal cerebral perfusion pressure (ΔCPP_opt_). Sample size of 416 patients unless otherwise specified. **A**, P_bt_O_2_ (n = 167): P_bt_O_2_ peaks at CPP_opt_ and remains stable at CPP values near CPP_opt_ (ΔCPP_opt_ +/−20 mm Hg) but decreases outside of this range. **B**, ICP: ICP decreases linearly with increasing ΔCPP_opt_ but does not continue to decrease once CPP values exceed CPP_opt_. **C**, ICP pulse amplitude: AMP decreases linearly with increasing ΔCPP_opt_, stabilizing at ΔCPP_opt_ of +10 to +40 mm Hg, and then continuing to decrease at ΔCPP_opt_ +40 mm Hg. AMP has an additional inflection point at ΔCPP_opt_ −40 mm Hg, where AMP begins to trend down. **D**, Compensatory Reserve Index RAP: RAP is stable from ΔCPP_opt_ of approximately −40 to 0 mm Hg, with 2 inflection points: at CPP_opt_, RAP begins to decrease with increasing ΔCPP_opt_; at ΔCPP_opt_, −40 mm Hg RAP begins to decrease with decreasing ΔCPP_opt_. **E**, PSI (n = 71): PSI decreases linearly with increasing ΔCPP_opt_. **F**, ICP slow wave amplitude: *Slow* is minimized at CPP_opt_, increasing as CPP values diverge from CPP_opt_. AMP, ICP pulse amplitude; CPP_opt_, optimal cerebral perfusion pressure; ICP, intracranial pressure; P_bt_O_2_, brain tissue oxygenation; PSI, pulse shape index; RAP, R-amplitude pressure.

P_bt_O_2_ data were available for 167 (1 excluded due to CPP availability for <6 hours) patients. The relationship between P_bt_O_2_ and ΔCPP_opt_ (Figure 1A), when aggregate data were considered, resembled a bell-shaped curve, with P_bt_O_2_ peaking and remaining stable at CPP values near CPP_opt_ but dropping once CPP diverged from CPP_opt_ by approximately +/−20 mm Hg. When data were weighted by patient, an ANOVA analysis revealed no significant difference in mean P_bt_O_2_ nor proportion of out-of-range P_bt_O_2_ at CPP values at, above, or below CPP_opt_ (*n* = 167, *P* = .49, *P =* .71).

### ICP and Derived Parameters

With aggregate data considered, ICP (Figure 1B), AMP (Figure 1C), RAP (Figure 1D), and PSI (Figure 1E) tended to decrease as ΔCPP_opt_ increased, with clear individual inflection points. ICP did not continue to decrease as CPP values exceeded CPP_opt_, with PSI and AMP only seeing a small drop once CPP values exceeded CPP_opt_ by 30 and 40 mm Hg, respectively. RAP remained stable at CPP values below CPP_opt_ but decreased linearly with ΔCPP_opt_ once CPP values exceeded CPP_opt_. Both RAP and AMP have concurrent inflection points at approximately −40 ΔCPP_opt_: at CPP values below this they decrease. Finally, *Slow* (Figure 1F) displays a U-shaped curve with a minimum at CPP values near CPP_opt_. In patient weighted data, the proportion of out-of-range ICP and mean AMP was significantly higher at CPP values below CPP_opt_ (*n* = 407, *P* < .001, *P* < .001) and significantly lower at CPP values above CPP_opt_ (*n* = 407*, P* < .001, *P* < .001). The mean RAP was significantly lower at CPP values above CPP_opt_ (*n* = 407, *P* < .001) but not significantly different at CPP values below CPP_opt_ (*n* = 407, *P* = .068). An ANOVA analysis revealed no significant differences in PSI below, at, or above CPP_opt_ (*n* = 71, *P* = .75). The same was true for the mean *Slow* (*n* = 407, *P* = .39).

### Patient Outcome and Mortality

Three hundred thirty two patients had GOS scores available 6 months postinjury. Eight of these had GOS 2, so this group was excluded because of poor sample size, leaving 324 patients. 207 patients (64%) had an unfavorable outcome, defined as a GOS ≤ 3, whereas the rest had a favorable outcome (GOS ≥ 4).

### Aggregate Relationships—Functional Outcome

**FIGURE 2.**
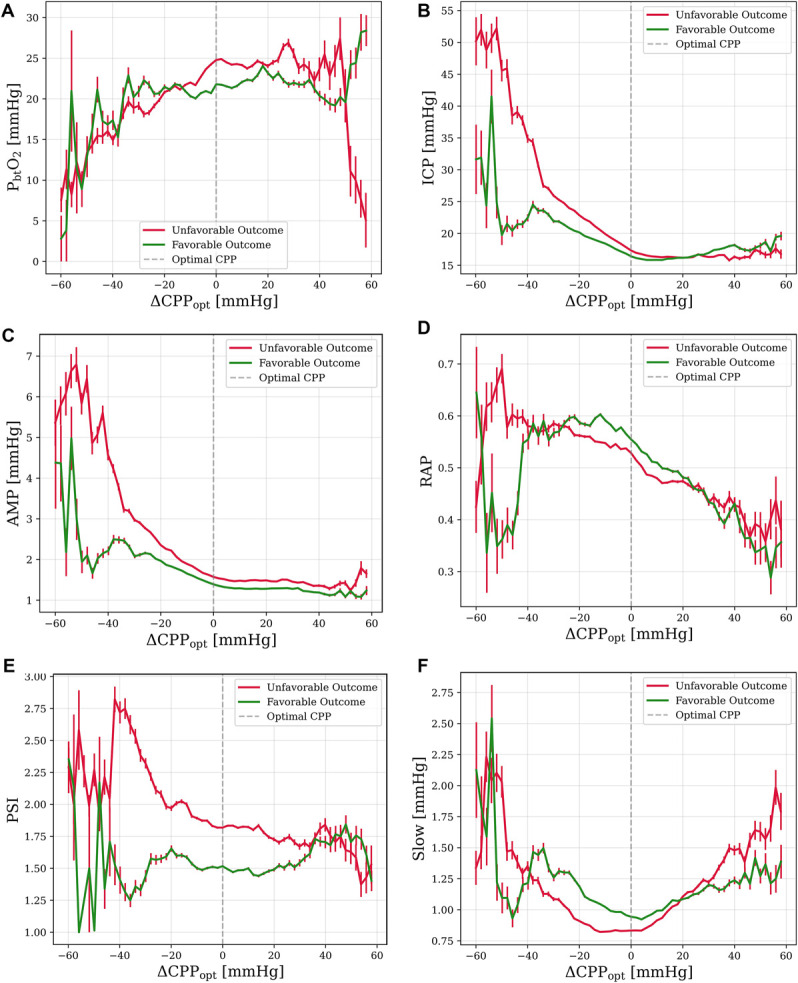
Mean values of intracranial parameters plotted against deviation from optimal cerebral perfusion pressure (ΔCPP_opt_), regarding outcome groups of GOS. Sample size of 324 patients unless otherwise specified. **A**, P_bt_O_2_ (n = 92): At CPP values below CPP_opt_, patients with an unfavorable outcome see drops in P_bt_O_2_. In those with a favorable outcome, P_bt_O_2_ drops at CPP values exceeding CPP_opt_. **B**, ICP: In both outcome groups, ICP decreases linearly with increasing ΔCPP_opt,_ but does not continue to decrease once CPP values exceed CPP_opt_. In patients with a favorable outcome, ICP begins to decrease as ΔCPP_opt_ drops below −40 mm Hg but continues to increase in patients with an unfavorable outcome. ICP is significantly higher in those with an unfavorable outcome (n = 324, *P* < .001). **C**, ICP pulse amplitude: In both outcome groups, AMP decreases linearly with increasing ΔCPP_opt,_ but does not continue to decrease once CPP values exceed CPP_opt_. In patients with a favorable outcome, AMP begins to decrease at ΔCPP_opt_ −40 mm Hg but continues to increase as ΔCPP_opt_ decreases in patients with an unfavorable outcome. AMP is significantly higher in patients with an unfavorable outcome (n = 324, *P* < .001). **D**, Compensatory Reserve Index RAP: In both outcome groups, RAP decreases as CPP exceeds CPP_opt._ In patients with an unfavorable outcome RAP is approximately stable from ΔCPP_opt_ of −60 to 0 mm Hg. In patients with a favorable outcome, RAP trends downward at as ΔCPP_opt_ goes below −30 mm Hg. RAP is significantly lower in patients with an unfavorable outcome (n = 307, *P* = .011). **E**, PSI: PSI decreases with increasing ΔCPP_opt._ PSI is significantly lower in patients with a favorable outcome (n = 64, *P* = .03). **F**, ICP slow wave amplitude: *Slow* is minimized at CPP_opt_ in both outcome groups. Mean *Slow* is significantly higher in patients with a favorable outcome (n = 324, *P* < .001). AMP, ICP pulse amplitude; CPP_opt_, optimal cerebral perfusion pressure; GOS, Glasgow Outcome Scale; ICP, intracranial pressure; P_bt_O_2_, brain tissue oxygenation; PSI, pulse shape index; RAP, R-amplitude pressure.

In plots of aggregate patient data, P_bt_O_2_ (Figure 2A) increased at CPP values above CPP_opt_ in patients with an unfavorable outcome, but not in patients with a favorable outcome; At CPP values below CPP_opt_, P_bt_O_2_ decreased in patients with an unfavorable outcome but remained stable in patients with a favorable outcome. ICP (Figure 2B), AMP (Figure 2C), and RAP (Figure 2D) demonstrated comparable relationships in aggregate data in both outcome groups. However, at CPP values lower than 40 mm Hg below CPP_opt_, outcome groups diverged, with patients with an unfavorable outcome seeing a continued rise in parameter value, whereas those with a favorable outcome saw a marked drop in parameter value. Pulse shape index (Figure 2E) remained stable in patients with a favorable outcome but decreased linearly with increasing CPP values in those with an unfavorable outcome. *Slow* (Figure 2F) retained the aggregate U-shaped curve between outcome groups. The mean ICP, AMP, *Slow*, PRx, RAP, and PSI were predictors of outcome, with favorable outcome patients having lower ICP (n = 324, *P* < .001), AMP (n = 324, *P* = .021), and PRx (n = 324, *P* < .001) and higher *Slow* (n = 324, *P* < .001), PSI (n = 64, *P* = .02), and RAP (n = 307, *P* = .011). The mean P_bt_O_2_ (n = 92, *P* = .35) and MAP (n = 324, *P* = .58) were not associated with outcome.

### Aggregate Relationships—Mortality

**FIGURE 3.**
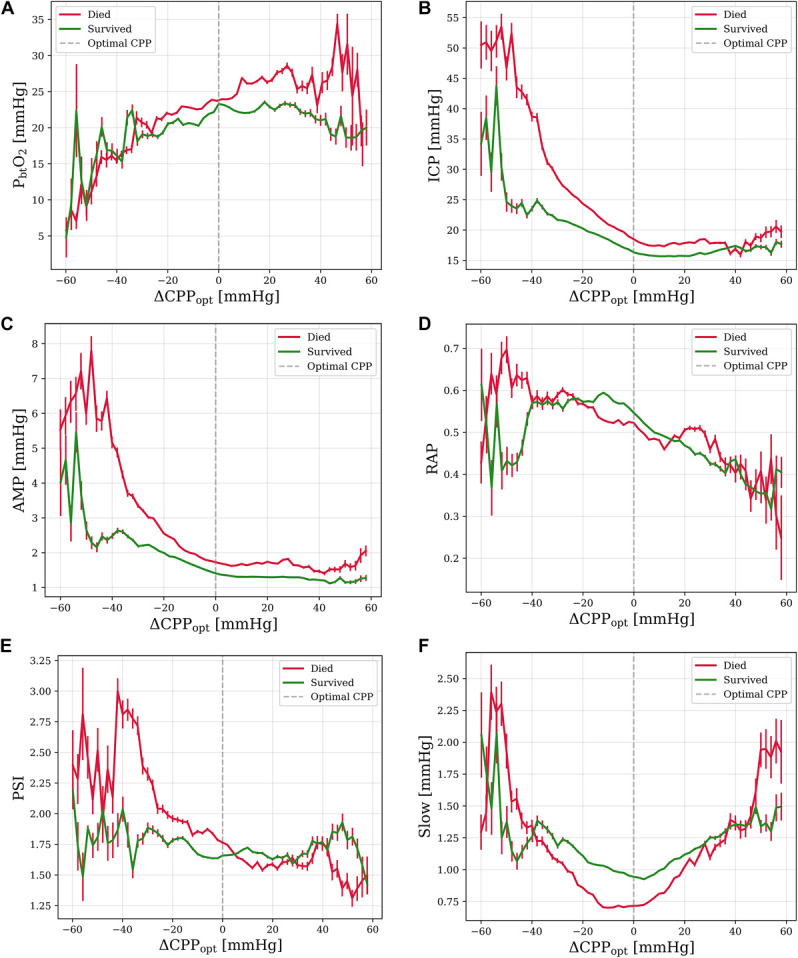
Mean values of intracranial parameters plotted against deviation from optimal cerebral perfusion pressure (ΔCPP_opt_), regarding mortality. Sample size of 324 patients unless otherwise specified. **A**, P_bt_O_2_ (n = 92): At CPP values below CPP_opt_, patients who died demonstrate drops in P_bt_O_2_. In surviving patients, P_bt_O_2_ drops at CPP values exceeding CPP_opt_. **B**, ICP: In both outcome groups, ICP decreases linearly with increasing ΔCPP_opt,_ but does not continue to decrease once CPP values exceed CPP_opt_. In patients who survived, ICP does not increase at CPP values below ΔCPP_opt_ −40 mm Hg but continues to increase as ΔCPP_opt_ decreases in patients who died. ICP is significantly lower in surviving patients (*P* < .001). **C**, ICP pulse amplitude: In both outcome groups, AMP decreases linearly with increasing ΔCPP_opt,_ past CPP_opt_. In patients who died, AMP continues to increase as ΔCPP_opt_ decreases, but stabilizes at ΔCPP_opt_ of approximately −20 mm Hg in surviving patients. AMP is significantly higher in patients who died (*P* < .001). **D**, Compensatory Reserve Index RAP: In patients who died, RAP decreases linearly as ΔCPP_opt_ increases. In surviving patients, RAP increases with increasing ΔCPP_opt_ from −60 to −10 mm Hg and then begins decreasing at CPP_opt_. RAP is significantly lower in patients who died (*P* = .042). **E**, PSI: In both outcome groups, PSI decreases with increasing ΔCPP_opt_. **F**, ICP Slow Wave Amplitude: *Slow* is minimized at CPP_opt_ in both outcome groups. Mean *Slow* is significantly higher in patients who survived (*P* < .001). AMP, ICP pulse amplitude; CPP_opt_, optimal cerebral perfusion pressure; ICP, intracranial pressure; P_bt_O_2_, brain tissue oxygenation; PSI, pulse shape index; RAP, R-amplitude pressure.

Aggregate plotting of parameters vs ΔCPP_opt_ stratified by mortality (Figure 3) resembled that of functional outcome (Figure 2). A small difference was observed in plots of ICP (Figure 3B), in which ICP did not decrease in surviving patients at CPP values 40 or more below CPP_opt,_ but rather remained stable as CPP values dropped further below CPP_opt_. The mean ICP, AMP, PRx, *Slow*, and RAP were predictors of mortality, with surviving patients having lower mean ICP (n = 324, *P* < .001), AMP (n = 324, *P* < .001), and PRx (n = 324, *P* < .001) and higher mean RAP (n = 307, *P* = .042) and *Slow* (n = 324, *P* < .001). MAP (n = 324, *P* = .26) and P_bt_O_2_ (*n* = 92, *P* = .45) were not associated with mortality.

### Treatment Thresholds and Outcome

**FIGURE 4.**
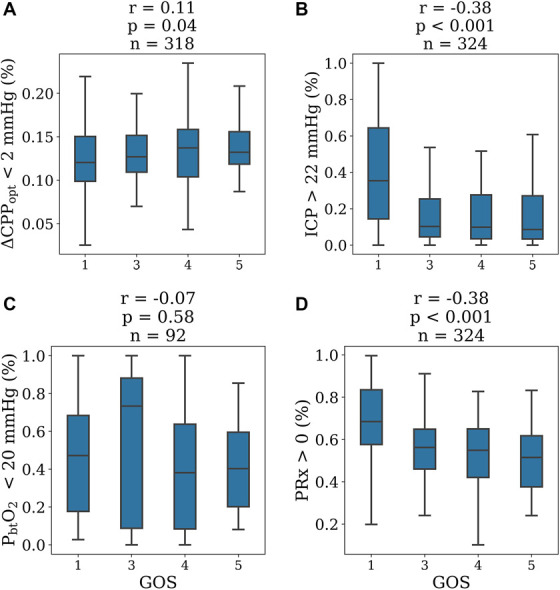
Box-and-whisker plots showing proportion of out-of-range values (with the exception of **A**: time at CPP_opt_) against GOS. Sample size shown inside the plots, with statistical significance test results above. **A**, Proportion of time at CPP_opt_ (+/−2 mm Hg from calculated CPP_opt_). **B**, Proportion of time out of ICP treatment threshold (>22 mm Hg). **C**, Proportion of time out of P_bt_O_2_ treatment threshold (<20 mm Hg). **D**, Proportion of time with PRx >0. Proportion of time with PRx >0 and ICP >22 mm Hg were found to be negatively correlated with GOS (r = −0.38, *P* < .001 for both), whereas time near CPP_opt_ was found to be positively correlated with GOS (r = 0.11, *P* = .04). CPP_opt_, optimal cerebral perfusion pressure; GOS, Glasgow Outcome Scale; ICP, intracranial pressure; P_bt_O_2_, brain tissue oxygenation; PRx, pressure reactivity index; PSI, pulse shape index.

The mean proportion of out-of-range ICP and PRx was significantly higher (Figure 4) in dying patients and patients with an unfavorable outcome (n = 324, *P* < .001). The mean proportion of CPP values near CPP_opt_ was significantly lower in dying patients (n = 318, *P* = .04), but not significantly different between the unfavorable and favorable outcome groups (n = 318, *P* = .08). The mean proportion of out-of-range P_bt_O_2_ was not significantly associated with mortality or outcome (n = 92, *P* = .86, *P* = .26, respectively). Regarding correlations with GOS, the mean proportion of out-of-range ICP and PRx was moderately negatively correlated with increasingly worse outcome (n = 324, r = −0.38, r = −0.38, *P* < .001, *P* < .001). Proportion of CPP values near CPP_opt_ was weakly correlated with improving outcome (n = 318, r = 0.11, *P* < .04). Proportion of out-of-range P_bt_O_2_ was not significantly correlated with outcome (r = −0.07, *P* = .58, n = 92).

## DISCUSSION

### Key Results

Our results provide confirmation^18^ that CPP_opt_ is a physiologically meaningful value given the demonstration of clear relationships with other physiological variables including ICP, P_bt_O_2_ as well as RAP, AMP, *Slow*, and PSI_._ Of particular support for this notion is that CPP values near CPP_opt_ serve as an inflection point for every measured parameter in aggregate data. This validates previous findings and confirms the important observation that in aggregate data, achieving CPP_opt_ is associated with optimized P_bt_O_2_. Another key finding of our work was that of the neuromonitoring variables studied, the achievement of acceptable ICP values was most strongly associated with outcome, suggesting that maintenance of acceptable ICP values should be the highest management priority for TBI treatment. It is important to confirm this finding in other patient cohorts, however, as the rigor with which normal ICP values were maintained in this patient cohort may confound this finding. Although PRx was an additional predictor of outcome, the ability of physicians to modify PRx is limited. However, it is tempting to investigate further whether joint ICP/PRx threshold would be even more efficient as a target for a treatment. It is notable that we found no outcome differences related to out-of-range P_bt_O_2_ and that the correlation between better outcome and time near CPP_opt_ was weak (r = 0.11, Figure 4).

### Interpretation

Our results demonstrate that achievement of CPP_opt_ is associated with numerous physiological benefits, suggesting a benefit to targeting CPP_opt_ as a secondary priority. We suggest that targeting CPP_opt_ does not obviate the need for brain oxygen monitoring, however, because of idiosyncratic findings in individual patients and a noteworthy proportion of out-of-range P_bt_O_2_ values even when CPP_opt_ is achieved. However, targeting CPP_opt_ may have some utility in improving P_bt_O_2_ in circumstances where P_bt_O_2_ cannot be directly measured. Some benefits to raising CPP marginally (approximately 10 mm Hg) above CPP_opt_ were evident, including a progressive reduction in ICP and improved P_bt_O_2_. However, clinicians who raise CPP above 70 mm Hg must carefully monitor for pulmonary complications.^29,30^ Pushing patient's CPP above 70 mm Hg was historically aimed at ensuring adequate perfusion and reducing ICP by inducing autoregulatory vasoconstriction.^31^ However, because a randomized controlled trial associated CPP values >70 mm Hg with pulmonary complications and worse outcomes, it is generally recommended to maintain CPP between 60 and 70 mm Hg.^32-34^ Our analysis of additional ICP-derived metrics (Figures 1-3) help to further confirm CPP_opt_ as physiologically significant, showing clear relationships between CPP_opt_ and RAP, AMP, and *Slow*. For RAP and AMP, aggregate results suggest interactions between intracranial volume, pressure, and compensatory reserve mechanisms. AMP and RAP (Figure 1C and 1D) each demonstrate inflection points at CPP values approximately 40 mm Hg below CPP_opt_, indicating that in aggregate data, it is at this point that the compensatory reserve is being depleted and AMP will begin to decrease with increasing mean ICP. A second inflection point at CPP_opt_ is observed in ICP, AMP, and RAP, an indication of functional compensatory reserve mechanisms. Differences in aggregate plots related to outcome and mortality (Figures 2 and 3) are likely a result of decompensation from hypoperfusion at low CPP values in patient with poor outcomes perhaps as a result of cytotoxic edema from energy failure. ICP (Figures 2B and 3B) is perhaps the clearest example of this, with ICP continuing to rise exponentially as CPP values drop in patients who died or had a poor outcome, while remaining stable or dropping in surviving patients or those with good outcome. It is concurrently observed in MAP plots (**Supplemental Digital Content 4** [http://links.lww.com/NEU/E676]) that MAP increases at low CPP levels, likely demonstrative of Cushing response. Surviving patients, or those with a favorable outcome, which did not exhibit this rise, may have not needed a Cushing response. Furthermore, vasopressors may not have been prescribed for these patients. Compared with other spectral components of the ICP signal, slow waves are relatively unexplored, although there have been studies which suggest they are correlated with sleep and respiration.^35,36^ Significantly, higher *Slow* in patients with good outcome, reported in the results, is supported by previous literature.^37^ Regarding Figure 1F, it is possible that as CPP diverges from CPP_opt_, hemodynamics diverge from a more resting state, and vasodilation and vasoconstriction are more likely to occur, stimulating *Slow*. PSI, a novel machine-learning–driven index, was shown to be physiologically significant, with differences relating to functional outcome. PSI also demonstrated a relationship with CPP_opt_ (Figure 1E). The decreasing trend in PSI with increasing Δ CPP_opt_ suggests that the waveform shape is more pathologically altered below CPP_opt_, which may stem from the concurrent change in the mean ICP (Figure 1B).

### Limitations

During care of the studied patients, ICP was aggressively maintained below the treatment threshold, but CPP_opt_ was not calculated or targeted. This may have confounded our results and provides strong rationale for repeating this analysis in patients in whom CPP_opt_ was targeted. The age of the data is also a limitation. At the time of the study, patients were managed to maintain ICP below 20 mm Hg per the contemporaneous Brain Trauma Foundation guidelines. These were revised in 2017 to recommend the current treatment threshold of 22 mm Hg.^27^

### Generalizability

The large cohort and diverse nature of the data, originating from 4 distinct trauma centers, allow these results to be generalizable to broader TBI research. As previously mentioned, this could be improved further with the inclusion and analysis of newer data, with treatment reflecting updated Brain Trauma Foundation guidelines. Finally, a recent study from Harder et al^38^ has demonstrated that findings from computed tomography scans indicative of elevated ICP can be used to improved prognosis after brain injury. Future studies on optimal or improved CPP would benefit from the incorporation of ICP-related features in their analyses.

## CONCLUSION

Our findings validate and extend our previous findings. We provide further evidence that CPP_opt_ is a physiologically meaningful value by revealing physiological inter-relationships in aggregate data between CPP_opt_ and other commonly measured parameters in brain-injured patients. Although many physiological parameters were improved when CPP_opt_ was achieved, the achievement of acceptable ICP values had the strongest association with outcome. Our data thus support an approach which prioritizes ICP-directed care with efforts to monitor and optimize P_bt_O_2_ and CPP_opt_ secondarily. Because individual patient-level analyses showed varied relationships between P_bt_O_2_ and CPP_opt_, and because brain hypoxia can persist even when CPP_opt_ is achieved, brain oxygen monitoring remains indicated even when CPP_opt_ can be targeted. Analysis of patients treated in a paradigm prioritizing the targeting of CPP_opt_ will provide important further insights into these physiological inter-relationships.

## Supplementary Material

SUPPLEMENTARY MATERIAL

## SUPPLEMENTAL DIGITAL CONTENT

**Supplemental Digital Content 1**. **Figure 1**. Demonstration of the well-understood relationship between intracerebral volume and intracranial pressure (ICP) is shown in a pressure-volume curve. With good compensatory reserve, ICP increases linearly with increased volume (RAP = 0). After continued increases in intracranial volume, ICP starts to increase exponentially, indicative of impaired compensatory reserve (RAP ∼1). Finally, with further volume increase, RAP begins to trend toward −1 cerebral hemodynamics become severely compromised and the patient is at risk of ischemia.^27^ CBF—cerebral blood flow. **Supplemental Digital Content 2**. **Table 1**. Recording periods for measured metrics in data set. **Supplemental Digital Content 3**. **Figure 2**. Distributions of measured and calculated variables across varying ΔCPP_opt_ values. **A**: P_bt_O_2_, **B**: ICP, **C**: ICP_slow_, **D**: PRx, **E**: RAP, **F**: ICP_amp._ Total number of patients with P_bt_o_2_ was 169, a subset of 431 patients with ICP and MAP. **Supplemental Digital Content 4**. **Figure 3**. Mean arterial blood pressure (MAP) plotted against deviation from optimal CPP in 431 patients.
