# Supplementary material for: Optimal Cerebral Perfusion Pressure in Brain Injury: Physiological Relationships and Outcome
Source: Neurosurgery. 2025 Apr 3;97(4):863–72. doi: 10.1227/neu.0000000000003411 (PMC12507320; doi:10.1227/neu.0000000000003411)

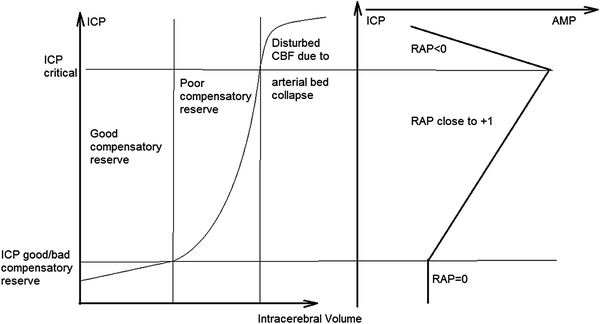
**Supplementary Figure 1.**

**Supplementary Table 1.**

| **Characteristic** | **Number of Patients** | **Total Duration of Measurements** | **Mean**  **Duration of Measurements** | **Maximum**  **Duration of Measurements** | **Minimum**  **Duration of Measurements** |
| --- | --- | --- | --- | --- | --- |
| Intracranial Pressure Monitoring | 431 | 2086 days | 5.0 days | 20.6 days | 7.0 hours |
| Focal Brain Oxygen Monitoring | 169 | 867 days | 5.1 days | 20.2 | 8.9 hours |
| Arterial Blood Pressure Monitoring | 431 | 2137 days | 5.1 days | 21.0 days | 7.1 hours |

**Supplementary Figure 2.**


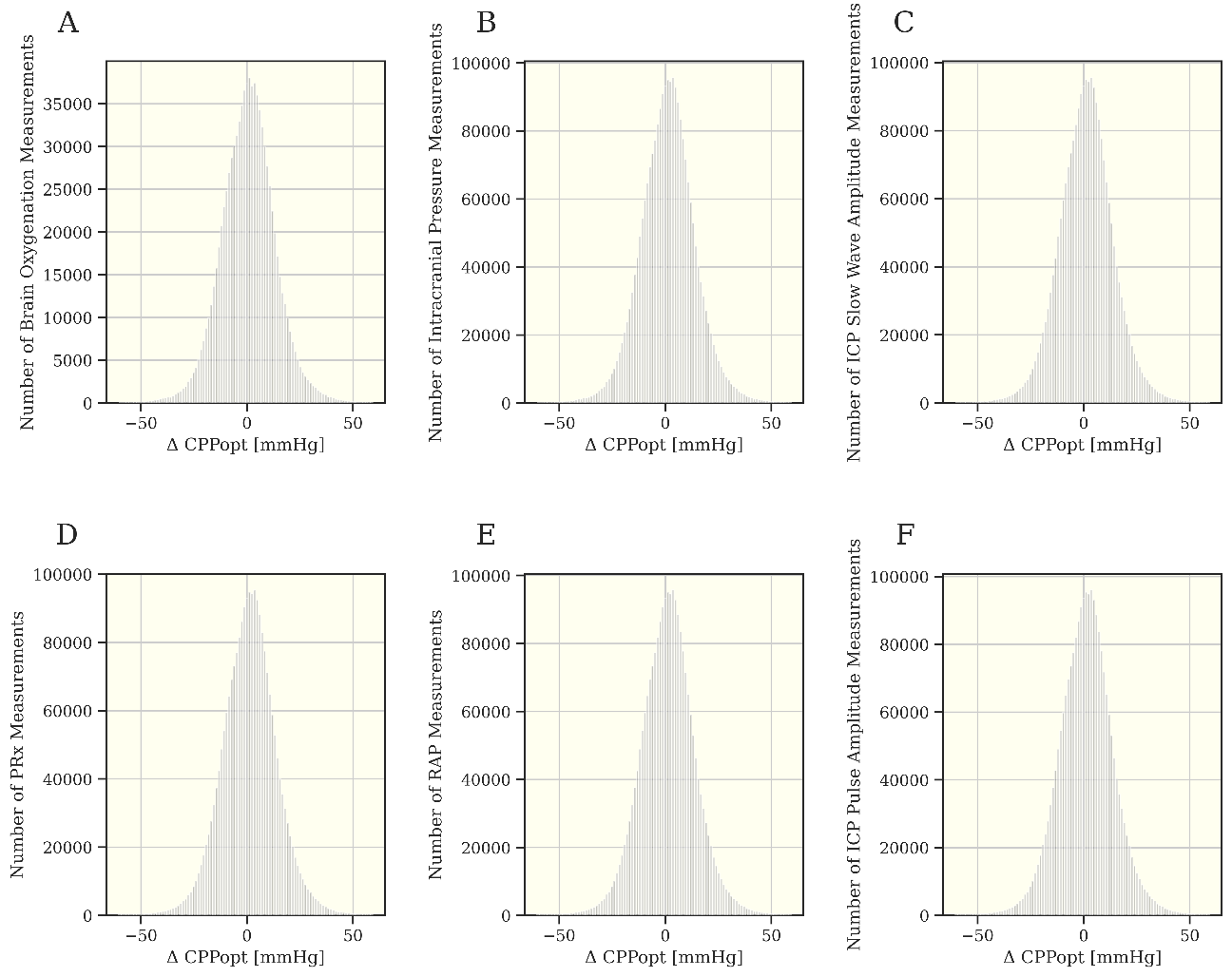


**Supplementary Figure 3.**


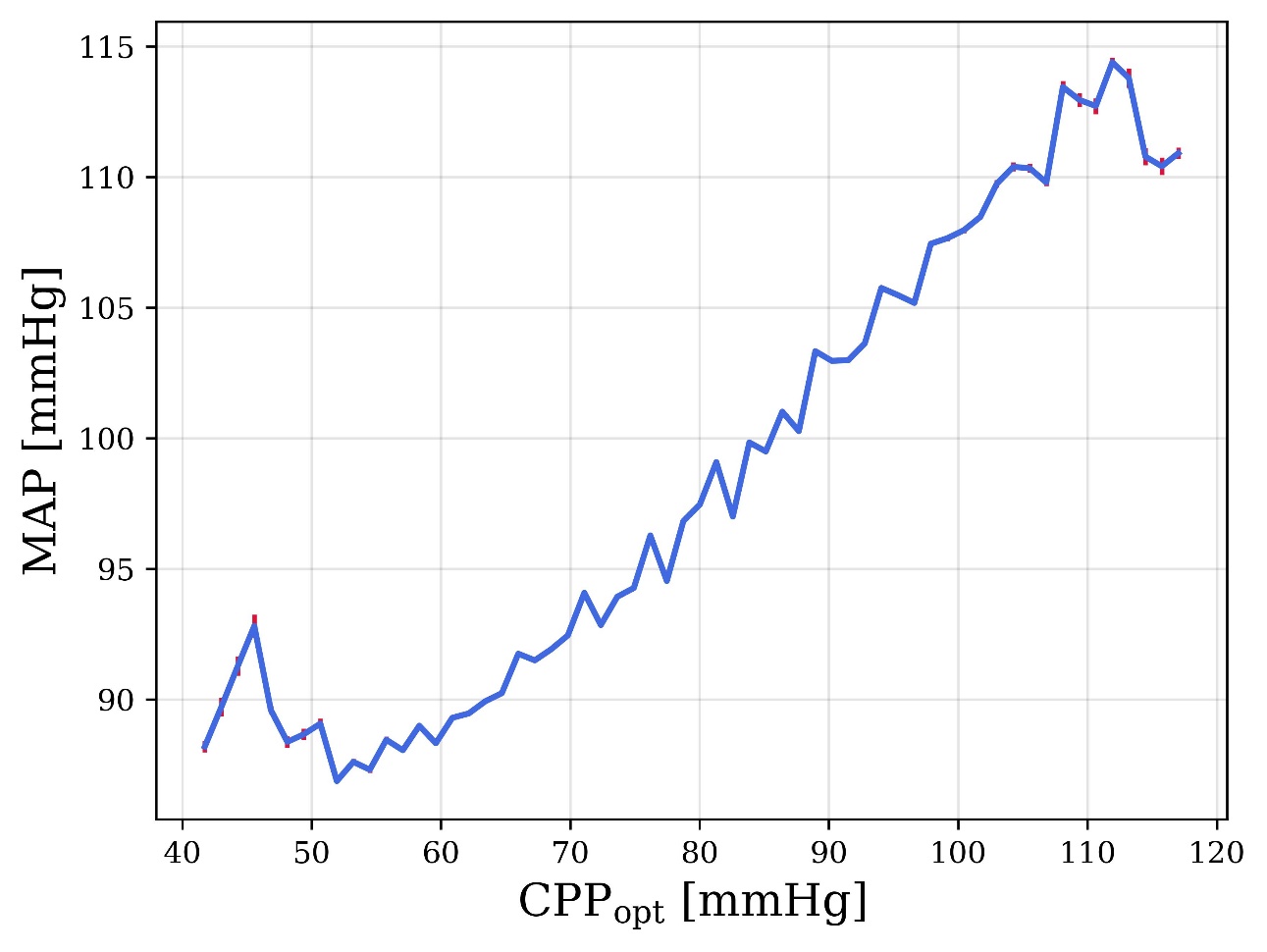

Supplement: SUPPLEMENTARY MATERIAL [file neu-97-863-s001.docx]
